# Distinct Morphokinetic Signature of Human Embryos with Chromosomal Mosaicism

**DOI:** 10.3390/genes16111388

**Published:** 2025-11-18

**Authors:** Margarita Ruseva, Sophia Zlatanova, Stefka Nikolova, Teodora Tihomirova, Dimitar Parvanov, Rumiana Ganeva, Maria Handzhiyska, Jinahn Safir, Dimitar Metodiev, Maria Pancheva, Maria Serafimova, Blaga Rukova, Rada Staneva, Georgi Stamenov, Savina Hadjidekova

**Affiliations:** 1 Research Department, Nadezhda Women’s Health Hospital, 1373 Sofia, Bulgaria; margarita.ruseva@gmail.com (M.R.); rum.ganeva@gmail.com (R.G.); jinahn.safir@gmail.com (J.S.); 2 Embryology Department, Nadezhda Women’s Health Hospital, 1373 Sofia, Bulgariastefka.v.nikolova@gmail.com (S.N.); 3 Obstetrics & Gynecology Department, Nadezhda Women’s Health Hospital, 1373 Sofia, Bulgaria; t.tihomirova@mail.bg (T.T.);; 4 Pathology Department, Nadezhda Women’s Health Hospital, 1373 Sofia, Bulgaria; 5 Genetics Department, Nadezhda Women’s Health Hospital, 1373 Sofia, Bulgariasvhadjidekova@medfac.mu-sofia.bg (S.H.); 6 Department of Medical Genetics, Faculty of Medicine, Medical University of Sofia, 1431 Sofia, Bulgaria

**Keywords:** preimplantation genetic testing for aneuploidy (PGT-A), next generation sequencing (NGS), mosaic embryos, time-lapse monitoring (TLM), morphokinetic parameters

## Abstract

Objectives: This study aimed to determine whether chromosomal mosaicism in blastocysts is associated with a distinct morphokinetic signature. Methods: Preimplantation genetic testing for aneuploidy (PGT-A) was performed on 182 human embryos via trophectoderm biopsy on day 5 and analyzed by next-generation sequencing. Embryos were classified as euploid (n = 55), mosaic (n = 39: 21 low-grade, 18 high-grade), or aneuploid (n = 88), of which 18 with concurrent mosaicism. Prior to biopsy, embryos were cultured in a time-lapse system (EmbryoScope), and 12 morphokinetic parameters were assessed, including pronuclei fading (tPNf), cleavage times (t2–t9), morula formation (tM), blastulation start (tSB), and full blastocyst formation (tB). These parameters were compared according to ploidy status. Results: Patients with euploid and mosaic embryos were comparable in terms of maternal age, ART indication and embryo quality (*p* > 0.05). In contrast, aneuploid embryos were obtained from older patients and had lower morphological grades. Mosaic embryos showed delayed tPNf (24.8 ± 6.5 vs. 22.8 ± 2.3 h, *p* = 0.03) and t2 (27.6 ± 6.6 vs. 25.4 ± 2.5 h, *p* = 0.02) compared to euploid embryos, mainly attributable to low-grade mosaic embryos. Whole-chromosome mosaicism, but not segmental mosaicism, was associated with delayed embryo development at several intermediate cleavage time points (t3, t4, t6, t7 and t9). Aneuploid embryos showed significant delays at later stages versus euploid embryos, particularly aneuploid embryos with mosaicism at t7 (56.6 ± 8.3 vs. 52 ± 5.6 h, *p* = 0.02), t8 (59.1 ± 9.6 vs. 54.8 ± 6.7 h, *p* = 0.04), tM (90.3 ± 7.7 vs. 83.6 ± 8.2 h, *p* = 0.006) and tB (113.0 ± 11.6 vs. 106.6 ± 8.9 h, *p* = 0.03). Conclusions: Mosaic embryos exhibit delays in early development (tPNf, t2) but reach later morphokinetic milestones at rates similar to euploid embryos. In contrast, aneuploid embryos, especially those with mosaicism, exhibit marked developmental delays at later stages (t7, t8, tM, tB).

## 1. Introduction

Genetic mosaicism is defined as the presence of two or more cell lines in an individual with a different genetic makeup, derived from a single zygote [1]. This phenomenon arises due to post-zygotic mutations of different type and size such as single nucleotide variants (SNVs), copy number variants (CNVs), and chromosomal abnormalities. It can also be classified according to the type of tissue affected (somatic, gonadal) which is indicative of the risk for the progeny, and the extent to which that tissue is affected [2].

One of the most significant aspects of embryonic development is the occurrence of chromosomal mosaicism, where an embryo contains chromosomally distinct cells. It is usually attributable to mitotic errors during early cell division, resulting in a mixture of karyotypically normal and abnormal cells. It can persist throughout post-implantation developmental stages and has been detected in various fetal and adult organs [3]. Mosaic embryos are often seen as compromised in terms of their developmental potential, with concerns over the risk of failed implantation, pregnancy loss, and congenital disorders in live-born infants [4,5]. However, studies suggest that they may possess a remarkable potential for chromosomal errors compensation, which is the ability to restore their genetic composition through the elimination of abnormal cells, or the balancing of cell dysfunction by normal cells [6]. Depending on local regulations, mosaicism thresholds and the chromosome affected, mosaic embryos are considered for transfer [7]. There is a growing body of evidence of healthy live births after their use in assisted reproduction [7,8,9].

It is undisputed that the genetic makeup of an embryo is a major determinant of reproductive success in both naturally conceived and in IVF pregnancies. The field of assisted reproduction is constantly searching for reliable and noninvasive technologies to predict the embryo ploidy status and prioritize embryos for transfer, eliminating risks associated with biopsy or termination of pregnancy with genetic abnormality.

Embryonic development is a highly dynamic and intricate process that involves a series of orchestrated, sequential cellular events and interactions, which can be investigated in detail through the use of time-lapse microscopy (TLM) systems [10]. Morphokinetics is devoted to the study of the timing and sequence of cellular divisions and morphological changes that occur during early embryogenesis, particularly during the stages of embryo cleavage. Continuous monitoring of these processes can provide valuable insights into embryo quality, developmental potential, and potentially the likelihood of a successful pregnancy. Using advanced imaging techniques to track the timing of cell divisions and other morphokinetic parameters could improve the understanding of the behavior and developmental potential of mosaic embryos, helping clinicians assess the likelihood of abnormal cell elimination and optimize embryo selection strategies for assisted reproductive technologies. This, in turn, could improve the success rates of ART procedures and expand the possibilities for embryo transfer, even in cases previously deemed unsuitable due to mosaicism.

In this context, the present study investigated whether embryos containing mosaic cell lines display a distinct morphokinetic signature compared to karyotypically normal embryos.

## 2. Materials and Methods

### 2.1. Ethical Approval

The study was conducted in accordance with the ethical standards outlined in the Declaration of Helsinki and approved by the Institutional Ethics Committee of Nadezhda Women’s Health Hospital (protocol code 7A/01.09.2023). All patients provided informed consent to participate in the study and for the publication of the anonimised results.

### 2.2. Patients

This retrospective observational cohort study was conducted at a private in vitro clinic from November 2023 to September 2024. Female patients aged 23 to 47 years (average 36.0 ± 5.1 years) undergoing assisted reproduction treatment were recruited. Embryos obtained after intracytoplasmic sperm injection (ICSI) were cultured in a time-lapse incubator (EmbryoScope™, Unisense, Aarhus, Denmark). Blastocysts were assessed according to the Gardner embryo grading system, assigning separate quality scores to the inner cell mass (ICM) and trophectoderm (TE) [11]. In total, 182 blastocysts from 147 couples with determined ploidy status and whole video were included.

### 2.3. Oocyte Retrieval, Embryo Culture

Oocyte retrieval was performed by transvaginal ultrasound-guided follicular aspiration 36 h after hCG administration. ICSI was performed on metaphase II oocytes approximately 4 h after oocyte retrieval. Following fertilization, embryos were placed in the time-lapse incubator and cultured until day 5 (D5) or 6 (D6) under 5% oxygen concentrations and variable carbon dioxide concentration to maintain pH levels between 7.2 and 7.4. Global^®^ total^®^ LP single-step medium for uninterrupted embryo culture (LifeGlobal, Guilford, CT, USA) was used. The EmbryoScope™ was programmed to acquire images of each embryo every 10 min through 11 different focal planes.

### 2.4. Evaluation of Time-Lapse Imaging and Morphokinetic Parameters

Two independent, well-trained embryologists rewatched the time-lapse videos in EmbryoViewer (Unisense, Aarhus, Denmark) and annotated developmental events and cleavage timepoints. The sperm microinjection was designated as time zero (t0), and computer software was used to calculate the timing in hours of the following events from fertilization: until the pronuclear fading (tPNf), cleavage to 2, 3, 4, 5, 6, 7, 8, 9 cells (t2, t3, t4, t5, t6, t7, t8 and t9 respectively), embryo formation into a morula (tM), the start of a cavity forming (tSB) and time of full blastocyst formation (tB).

### 2.5. NGS-Based Preimplantation Genetic Testing for Chromosomal Abnormalities (PGT-A)

Trophectoderm biopsies were performed on blastocyst-stage embryos (d5 or d6). Biopsied cells underwent DNA extraction which was then assessed for purity and quantified. Following library preparation using the EmbryoMap Sample Prep kit (Vitrolife, Gothenburg, Sweden) and the MiSeq reagent kit v.3 (Illumina, San Diego, CA, USA), next-generation sequencing (NGS) was performed for numerical and structural chromosomal abnormalities with a resolution of 10 Mb on the MiSeq platform (Illumina, USA). Data analysis was performed using the EmbryoMap software v.0.3.1-a (Vitrolife, Gothenburg, Sweden). Embryos with aneuploid percentage below 20% were classified as euploid, those with aneuploidy rate between 20% and 80% were categorized as mosaic, and embryos with >80% aneuploidy were classified as aneuploid. Aneuploid embryos were further subdivided into those displaying concurrent mosaicism and those that were purely aneuploid. All subsequent analyses were performed to identify differences in developmental timing between embryos with distinct ploidy statuses.

### 2.6. Statistical Analysis

Baseline characteristics were summarized using descriptive statistics and are presented as mean ± SD for continuous data or count (percentage) for categorical data. To assess the consistency of time-lapse annotations between embryologists, inter-observer reliability was evaluated using the intraclass correlation coefficient (ICC). Two independent embryologists annotated all embryo development videos, blinded to ploidy status (as genetic testing was performed after the completion of time-lapse monitoring). The ICC was calculated using a two-way random effects, absolute agreement, average-measures, corresponding to ICC(2,k) according to standard convention. Comparisons of morphokinetic parameters between groups with different ploidy status were performed using unpaired t-test or Mann–Whitney U-test and one-way ANOVA or Kruskal–Wallis test according to normality, and reported as mean ± SD or median [interquartile range], as appropriate. All data were analyzed using the SPSS software (version 27.0, IBM Corporation, Armonk, NY, USA), and *p* < 0.05 was used as the predetermined significance cutoff.

## 3. Results

### 3.1. Study Population

**Figure 1:**
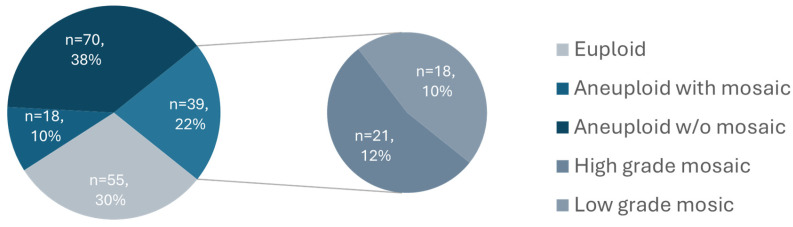
Distribution of trophectoderm biopsy ploidy results as determined by NGS.

Of the 213 ICSI cycles performed, 147 resulted in blastocysts with full video captured and were therefore included in the study. Of these, 117 couples contributed a single embryo, 25 couples contributed two embryos, and 5 couples contributed three embryos. Overall, 20.4% of couples (30/147) contributed more than one embryo. Among them, 19 contributed embryos which were all classified in the same ploidy category, while 11 (7.5%) contributed embryos that fell into different ploidy categories. The average maternal age was 36.0 years (95% CI 35.3–36.8). In total, 182 blastocysts cultured in a time-lapse incubator were biopsied and screened for aneuploidy by NGS. The baseline patient and embryo characteristics are summarized by ploidy status in Table 1. Representative images illustrating the three-tier morphological grading system for the inner cell mass (ICM) and trophectoderm (TE) are shown in Figure S1. The euploidy rate in our cohort was 30%, the aneuploidy rate was 48%, and mosaicism accounted for 22% of the tested blastocysts (Figure 1).

**Figure 2:**
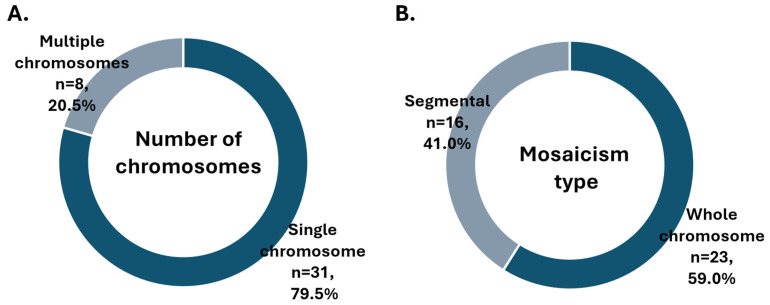
Distribution of mosaicism results by (**A**) number of chromosomes involved and (**B**) extent of chromosome affected.

**Figure 3:**
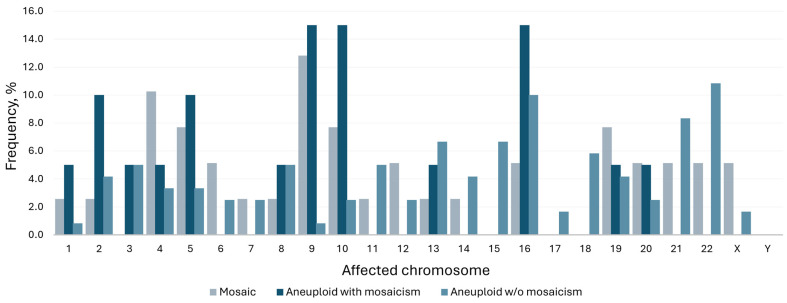
Frequency of affected chromosomes by ploidy status.

Looking in more detail into the subtypes of mosaicism, in the majority of cases, a single chromosome or segment was duplicated or missing (Figure 2A), and usually, the entire chromosome was affected (Figure 2B). As depicted on the bar chart, mosaicism affected larger chromosomes more often (chromosomes 4, 5 and 9 being most common), while aneuploidy was more prevalent among smaller chromosomes (16, 21 and 22) (Figure 3). To further explore whether chromosomal constitution influences developmental kinetics, we compared the timing of key morphokinetic milestones among ploidy groups.

Navy and grey bars denote mosaicism frequency; light blue represents aneuploidy rate.

Since two independent embryologists annotated the developmental timepoints, we assessed the consistency in annotations between them. The inter-observer reliability was determined across all morphokinetic timepoints. Using a two-way random-effects, absolute agreement model [ICC(2,k)], ICC values ranged from 0.89 to 0.97, with the lowest agreement observed for t5 and the highest for tB (Table S1). These results indicate a high level of reproducibility and confirm the robustness of the manual time-lapse annotations.

### 3.2. Mosaic vs. Euploid

**Figure 4:**
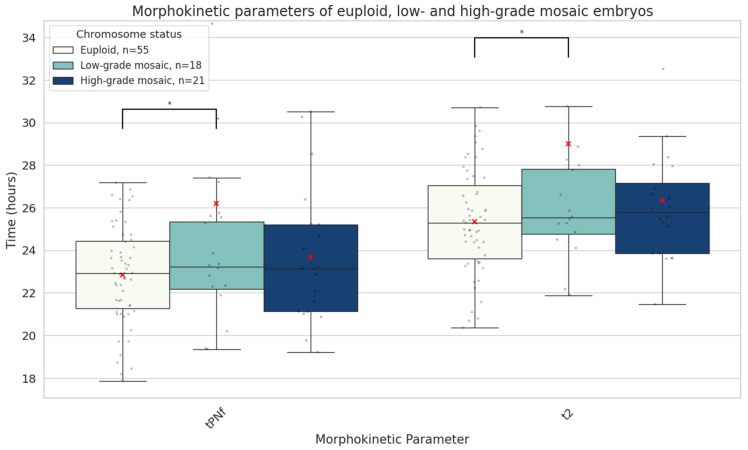
Boxplots representing the times in hours it takes euploid (n = 55), low-grade mosaic (<50% aneuploidy rate, n = 18) and high-grade mosaic (≥50% aneuploidy rate, n = 21) embryos until both pronuclei disappear (tPNf) and reach the 2-cell stage (t2). The red X demarcates the mean value. Asterisks denote significant differences vs. euploid rates.

There were no significant differences in patient age, ART indication, or embryo quality between the euploid and mosaic groups (Table 1). We found a significant difference in the developmental speed of mosaic (n = 39) and euploid (n = 55) blastocysts. Mosaic embryos were significantly slower in reaching the earliest two morphokinetic parameters compared to euploid ones: tPNf (24.8 ± 6.5 vs. 22.8 ± 2.3 h, mean absolute difference 2.0 h, *p* = 0.03) and t2 (27.6 ± 6.6 vs. 25.4 ± 2.5 h, mean absolute difference 2.2 h, *p* = 0.02). Upon detailed subgroup analysis, we discovered that in fact, low-grade mosaic embryos (containing up to 50% aneuploid cells) were exhibiting this pronounced delay in initial development, while high-grade mosaic embryos resembled euploid embryos in terms of initial developmental speed (Figure 4).

**Figure 5:**
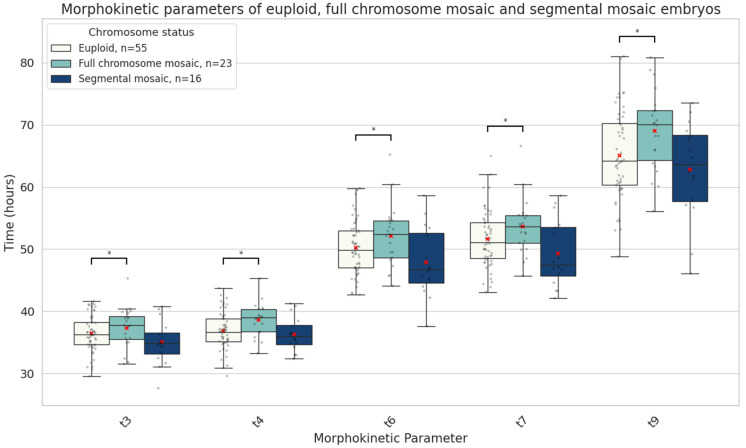
Boxplots representing the times in hours it takes euploid (n = 55), full chromosome mosaic (n = 23) and segmental mosaic (n = 16) embryos until they reach 3-cell (t3), 4-cell (t4), 6-cell (t6), 7-cell (t7) an 9-cell (t9) stages. The red X demarcates the mean value. Asterisks denote significant differences vs. euploid rates.

Further subanalysis revealed that when mosaicism involved an entire chromosome rather than a chromosome segment, embryos exhibited developmental delay at the intermediate time points (t3, t4, t6, t7, and t9) (Figure 5).

### 3.3. Aneuploid with Mosaic vs. Euploid

**Figure 6:**
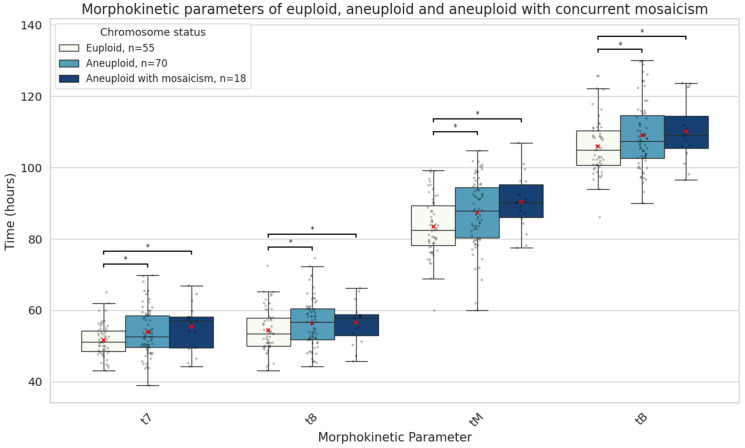
Boxplots representing the times in hours until the 7-cell stage, 8-cell stage, morula, and full blastocyst formation of euploid embryos (n = 55), aneuploid with (n = 18), and aneuploid embryos without concurrent mosaicism (n = 70). The red X demarcates the mean value. Asterisks denote significant differences vs. euploid rates.

Patients with aneuploid embryos were significantly older, and their embryos had lower morphological grades compared to those with euploid embryos (Table 1). Aneuploid embryos, particularly those with mosaic cell lines, showed significant delays in later developmental stages compared to euploid embryos. The aneuploid embryos with mosaicism were the slowest to reach the 7-cell (57.2 [11.0] vs. 51.3 [6.0] h, median absolute difference 5.9 h, *p* = 0.03), 8-cell (58.3 [11.0] vs. 53.5 [9.0] h, median absolute difference 4.8 h, *p* = 0.04), morula (90.2 [11.0] vs. 82.5 [12.0] h, median absolute difference 7.7 h, *p* = 0.006), and blastocyst stages (110.1 [17.0] vs. 104.9 [10.0] h, median absolute difference 5.2 h, *p* = 0.02) (Figure 6).

## 4. Discussion

Mosaicism identified through PGT-A remains a topic of active debate in reproductive medicine. While mosaicism is frequently observed in healthy adults, suggesting it may reflect normal biological variation rather than a pathological state, even low-level chromosomal abnormalities in embryos can lead to developmental arrest or miscarriage [12,13]. Studies have shown that mosaic embryos exhibit altered global gene expression compared to their euploid counterparts [14], raising further concerns about their developmental potential. However, advances in single-cell sequencing have revealed that mosaicism is a common feature of early embryogenesis, with its incidence typically declining as development progresses [3,15]. There are very few documented cases of mosaicism persisting throughout fetal development until pregnancy resolution [16,17,18]. These findings suggest that mosaic embryos should not be discarded altogether, as they may still expand reproductive options for couples undergoing assisted reproduction.

PGT-A is typically performed at the blastocyst stage. Despite its clinical validation, PGT-A has been criticized for its inherent sampling bias due to the limited number of trophectoderm (TE) cells that are analyzed. This raises concerns regarding whether PGT-A truly reflects the genetic constitution of the inner cell mass (ICM)—the portion of the embryo that develops into the fetus. Moreover, studies have demonstrated poor concordance between repeated biopsies [19,20], raising concerns regarding the reliability of PGT-A results. Additional limitations of PGT-A include false-positive and false-negative rates of 2–3%, inability to detect chromosomal deletions/duplications smaller than the NGS platform resolution, or to identify balanced chromosomal rearrangements, polyploidy, haploidy, uniparental disomy (UPD), epigenetic abnormalities, or single-gene (monogenic) mutations. Given these limitations, confirmatory prenatal testing, such as amniocentesis, is recommended, especially following the transfer of a mosaic embryo.

Investigating the behavior and developmental potential of mosaic embryos has become a critical area of research in reproductive medicine. By using advanced imaging techniques to track the timing of cell divisions and other morphokinetic parameters, clinicians have been trying to optimize embryo selection strategies for assisted reproduction, including developing AI-assisted prediction models, which remain of limited clinical applicability [21,22,23]. Nevertheless, efforts in the field continue, including prediction of blastocyst formation, survival after thaw and ploidy status, which could in theory improve the understanding of the mechanisms governing these processes [24,25].

A significant challenge in the morphokinetics and PGT-A field lies in the disagreement between studies in terms of which parameters are of importance and their ‘normal’ ranges. This could be attributable to the lack of standardization in defining ploidy categories [26]. This is also evidenced by the substantial amount of variability in mosaicism rates reported among PGT-A providers. A recent large multicenter study found an inverse relationship between the rates of euploid and mosaic embryos, indicating that threshold definitions substantially impact embryo classification [27]. In our cohort, we observed a relatively high proportion of mosaic results from embryo biopsies (22%), with roughly equal distribution between low- and high-grade mosaics (Figure 1) and most frequently affecting large chromosomes (4, 5, and 9), concordant with data reported from large reference labs [5,9,28]. Our mosaic rate aligns closely with those reported from much larger datasets [9,29,30], while our euploid (30%) and aneuploid (48%) rates are somewhat higher and lower, respectively, likely reflecting cohort-specific factors such as maternal age, stimulation protocols, and laboratory methodologies. It is important to note that our analysis only included embryos that developed to the blastocyst stage and were biopsied. Had we included the entire cohort of zygotes, including those that failed to reach the blastocyst stage, the proportion of embryos with abnormal PGT-A results would likely have been higher.

Morphokinetic studies have provided mixed results regarding the developmental potential of mosaic embryos. Some evidence suggests that these embryos exhibit intermediate developmental profiles—more advanced than aneuploid but lagging behind euploid embryos [31]. Other reports suggest that early developmental delays are predictive of poorer embryo quality and lower implantation potential [32]. In our analysis, mosaic embryos demonstrated delayed development at early stages, particularly prior to the two-cell stage. However, beyond this point, they developed at a similar pace to euploid embryos. Notably, no significant differences were observed in morula or blastocyst quality between mosaic and euploid embryos (Table 1, Figure 4). In contrast, aneuploid embryos displayed disrupted timing and morphology throughout crucial later stages associated with genome activation and blastocyst formation (Table 1, Figure 5). Depending on the type of mosaic anomaly, however, developmental timelines could be affected. Our subgroup analyses suggest that whole-chromosome mosaicism has a more pronounced impact on early embryonic cleavage dynamics, potentially reflecting a greater disruption of cellular processes compared with segmental mosaicism (Figure 5). From a clinical perspective, the extent to which a chromosome is affected by mosaicism should be taken into account when evaluating such embryos for transfer.

Clinical outcomes following the transfer of mosaic embryos remain a heavily debated topic. While retrospective studies have reported lower implantation rates and increased miscarriage risk compared to euploid embryos [7,33], these findings may be confounded by patient selection bias. Specifically, mosaic embryos are often transferred in patients with limited or no euploid embryos available for transfer, suggesting that they are a subfertile population. More recent prospective non-selection trials have demonstrated that low-level mosaic embryos can yield clinical outcomes comparable to those of euploid embryos [9]. Furthermore, successful pregnancies and live births following the transfer of mosaic embryos have been increasingly documented since their first report a decade ago [5,7,34,35,36].

The developmental potential of mosaic embryos may be attributable to their capacity for compensating chromosomal errors, which is often somewhat misleadingly referred to as self-correction. Mosaic embryos have been shown to eliminate chromosomally abnormal cells through selective apoptosis and reduced cell proliferation, with slower cell cycles for the aneuploid cells [37,38,39,40]. This hypothesis is supported by recent studies in which expelled cells from embryos were found to contain chromosomal abnormalities in 85.7% of cases, regardless of the ploidy status of the biopsied TE cells [41]. Comprehensive animal data also supports selective cell expulsion as the predominant mechanism of mosaic rescue. These findings are consistent with our morphokinetic data for low-grade mosaics, which initially show delayed early cleavage but subsequently catch up with euploid embryos. While direct mechanisms for restoring euploidy, such as monosomic or trisomic chromosome rescue, have also been proposed as a correction mechanism for chromosomally abnormal cells [6,41], it is noteworthy that these processes can result in UPD, potentially still causing genetic disease if the chromosome contains imprinted genes or pathogenic recessive alleles. However, the incidence of UPD in human embryos is exceedingly rare (0–0.06%) [42,43]. Most evidence supports that ‘self-correction’ in mosaic embryos primarily occurs via the expulsion of abnormal cells. Studies suggest that embryos with mosaicism levels below 50% may successfully overcome chromosomal errors and develop into healthy offspring [26]. Our findings that low-grade mosaic embryos exhibit delays in early stages, but later accelerate to match the developmental speed of euploid embryos, support the notion that in embryos with low levels of mosaicism, selective expulsion of abnormal cells may overcome the imbalance in time.

Mosaic embryos demonstrate unique cellular dynamics during development. A study using immunofluorescent labelling of mitosis and apoptosis markers revealed that chromosomally unbalanced embryos show increased apoptosis and compensatory cell division—mechanisms likely aimed at offsetting the impaired proliferation of aneuploid cells [35]. These findings align with our own observation that mosaic embryos, while exhibiting early developmental delays, catch up in subsequent stages. We also observed that delayed early cleavage was specific to mosaic embryos; however, this did not translate into inferior morula or blastocyst morphology. On the contrary, aneuploid embryos showed abnormalities in both morula and blastocyst formation timing and quality throughout development. Only one study has reported morphokinetics of embryos with mosaicism with concurrent non-mosaic aneuploidy [36], and our results are similar in that these embryos exhibited delays in reaching the 8-cell stage and the blastocyst, which are key stages for genome activation and implantation.

Our findings contribute valuable data to the discussion on mosaic embryo development and clinical viability. Notably, we found that mosaic embryos can exhibit developmental patterns similar to those of euploid embryos, particularly beyond the initial cleavage stages. This may reflect the embryo’s intrinsic capacity for self-correction through the elimination of abnormal cells. However, our study is not without limitations. As with all studies relying on PGT-A, technical limitations—such as sampling bias, diagnostic resolution, and inherent error rates—must be acknowledged. Furthermore, the retrospective design and relatively small cohort size limit the generalizability of our conclusions. Including both D5 and D6 blastocysts in our analysis inherently introduces some heterogeneity in late developmental events; however, we considered this acceptable to preserve the full spectrum of developmental timing and avoid artificially constraining tB variability. Adjusting for or restricting by day of blastulation could have obscured meaningful biological differences between chromosomal groups.

Due to ethical constraints, we were limited to non-invasive imaging analyses and could not perform molecular staining of human embryos. Future studies using stem cell-derived embryo models or donated material under extended ethical approval could provide complementary mechanistic insights into the observed developmental delays.

Understanding how mosaic embryos evolve and identifying non-invasive indicators of their capacity for self-correction could enhance ART success rates and expand embryo transfer options, particularly in cases previously deemed unsuitable due to mosaicism.

## 5. Conclusions

In summary, chromosomal mosaicism in blastocysts is linked to distinct morphokinetic patterns during embryo development. Low-grade mosaic embryos begin their development notably later than euploid embryos. They exhibit delayed development at early stages, specifically in the time of pronuclei fading (tPNf) and the first cleavage (t2), but later accelerate to similar morphokinetic values as euploid embryos. The degree of chromosomal involvement in mosaicism also appears to influence developmental kinetics, with whole-chromosome but not segmental mosaicism, associated with delayed intermediate time points. In contrast, aneuploid embryos with mosaicism show developmental delays at later stages (t7, t8, tM, and tB), which are associated with genome activation and implantation and are more likely to be indicative of compromised embryo viability. These findings support the clinical reconsideration of low-grade mosaic embryos for transfer under appropriate genetic counselling and patient selection.

## Figures and Tables

**Table 1:** Baseline demographic and blastocyst characteristics classified by ploidy status.

Characteristic	Euploid (n = 55)	Mosaic (n = 39)	Aneuploid *w*/*o* Mosaic (n = 70)	Aneuploid with Mosaic (n = 18)	*p* Value
Maternal age, years	33.7 ± 4.4	34.6 ± 4.1	38.3 ± 5.3 *	37.3 ± 4.1 *	0.037 ^1^
ART indication					0.024 ^2^
Unexplained infertility, n (%)	21 (38.2%)	21 (53.8%)	46 (65.7%) *	12 (66.7%) *	
Tubal factor, n (%)	14 (25.5%)	4 (10.3%)	6 (8.6%) *	0 (0.0%) *	
Male factor, n (%)	17 (30.9%)	13 (33.3%)	15 (21.4%)	6 (33.3%)	
Other ^‡^, n (%)	3 (5.5%)	1 (2.6%)	3 (4.3%)	0 (0.0%)	
ICM morphology grade Grade A, n (%) Grade B, n (%) Grade C, n (%)	26 (47.3%) 28 (50.9%) 1 (1.8%)	17 (43.6%) 20 (51.3%) 2 (5.1%)	13 (18.6%) * 55 (78.6%) 2 (2.8%)	9 (50.0%) 8 (44.4%) 1 (5.6%)	0.026 ^2^
TE morphology grade Grade A, n (%) Grade B, n (%) Grade C, n (%)	3 (5.5%) 48 (87.3%) 4 (7.3%)	1 (2.6%) 33 (84.6%) 5 (12.8%)	2 (2.9%) 56 (80.0%) 12 (17.1%) *	1 (5.6%) 13 (72.2%) * 4 (22.2%) *	0.041 ^2^

Continuous data are presented as mean ± standard deviation and categorical data as count (percentage). *p* values were calculated using Kruskal–Wallis ^1^ or Chi-squared ^2^ tests. Values with asterisks denote statistical differences from the euploid embryo group based on pairwise comparisons using Bonferroni post hoc after adjusting for multiple comparisons. ART—assisted reproductive therapy, ICM—inner cell mass, TE—trophectoderm. ‡ genetic, endocrinological factors.

## Data Availability

The data supporting the findings is available at: https://doi.org/10.5281/zenodo.16318393.

## Supplementary Materials

The following supporting information can be downloaded at: https://www.mdpi.com/article/10.3390/genes16111388/s1, Figure S1: Representative images illustrating the three-tier grading system for the inner cell mass (ICM) and trophectoderm (TE); Table S1: Inter-observer reliability for time-lapse annotations; Video S1: Representative time-lapse video of an euploid embryo; Video S2: Representative time-lapse video of a low-grade mosaic embryo; Video S3: Representative time-lapse video of a high-grade mosaic embryo; Video S4: Representative time-lapse video of a fully aneuploid embryo; Video S5: Representative time-lapse video of an aneuploid embryo with concurrent mosaicism.

## Abbreviations

The following abbreviations are used in this manuscript:

PGT-A	Preimplantation Genetic Testing for Aneuploidy
NGS	Next-Generation Sequencing
TLM	Time-Lapse Monitoring
SNV	Single Nucleotide Variant
CNV	Copy Number Variant
ART	Assisted Reproductive Therapy
IVF	In Vitro Fertilization
ICSI	Intracytoplasmic Sperm Injection
ICM	Inner Cell Mass
TE	Trophectoderm
hCG	Human Chorionic Gonadotropin
t0	Time of Sperm Microinjection
tPNf	Time of Pronuclei Fading
t2–t9	Timepoints of Cleavage to 2–9 Cells
tM	Time of Morula Formation
tSB	Time of Start of Blastulation
tB	Time of Full Blastocyst Formation
D5/D6	Day 5/Day 6 of Embryo Development
SD	Standard Deviation
CI	Confidence Interval
UPD	Uniparental Disomy
Mb	Megabase
