# Supplementary material for: Distinct Morphokinetic Signature of Human Embryos with Chromosomal Mosaicism
Source: Genes (Basel). 2025 Nov 18;16(11):1388. doi: 10.3390/genes16111388 (PMC12652751; doi:10.3390/genes16111388)
Supplement: Supplementary file 1 [file genes-16-01388-s001.zip › Table S1 Interobserver reliability for timepoint annotations.pdf]

Table S1. Inter-observer reliability for time-lapse annotations

Morphokinetic timepoint	ICC(2,k)*	95% CI	Interpretation
tPNf	0.93	0.91–0.95	Excellent
t2	0.91	0.87–0.94	Excellent
t3	0.92	0.89–0.95	Excellent
t4	0.94	0.91–0.96	Excellent
t5	0.89	0.84–0.92	Good
t6	0.90	0.86–0.93	Excellent
t7	0.91	0.87–0.94	Excellent
t8	0.93	0.89–0.95	Excellent
t9	0.91	0.86–0.94	Excellent
tM	0.96	0.91–0.96	Excellent
tSB	0.95	0.92–0.97	Excellent
tB	0.97	0.93–0.98	Excellent

*ICC values were computed using a two-way random-effects, absolute agreement, average-measures model [ICC(2,k)]. Interpretation thresholds as follows: <0.5 poor, 0.5–0.75 moderate, 0.75–0.9 good, and >0.9 excellent reliability.
