# Supplementary figures and images for: Distinct Morphokinetic Signature of Human Embryos with Chromosomal Mosaicism
Source: Genes (Basel). 2025 Nov 18;16(11):1388. doi: 10.3390/genes16111388 (PMC12652751; doi:10.3390/genes16111388)

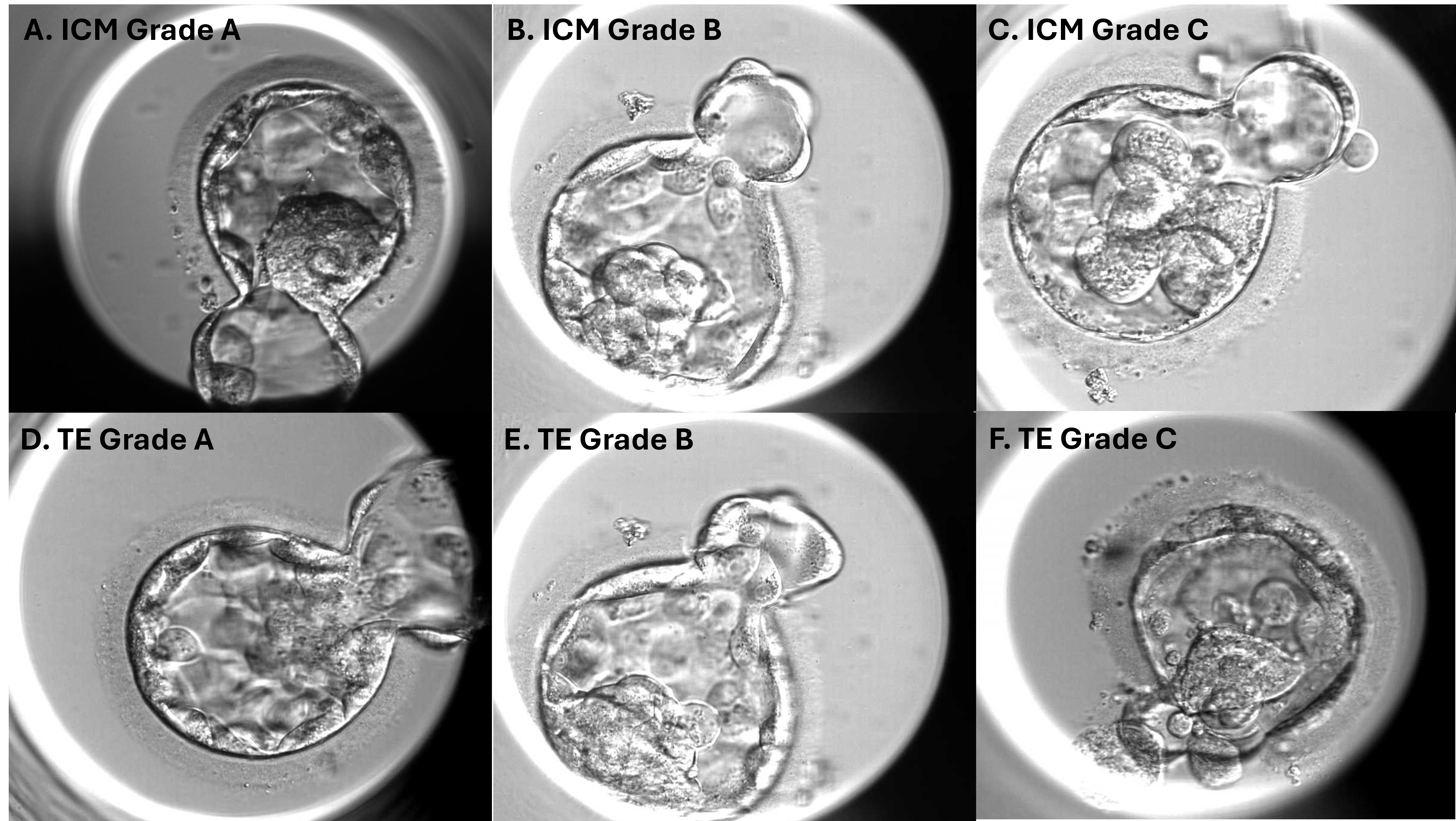

Supplement: Supplementary file 1 [file genes-16-01388-s001.zip › Figure S1.png]
